# Isotopic Evidence That Dragonflies (*Pantala flavescens*) Migrating through the Maldives Come from the Northern Indian Subcontinent

**DOI:** 10.1371/journal.pone.0052594

**Published:** 2012-12-20

**Authors:** Keith A. Hobson, R. Charles Anderson, David X. Soto, Leonard I. Wassenaar

**Affiliations:** 1 Environment Canada, Saskatoon, Saskatchewan, Canada; 2 Manta Marine Pvt Ltd, Malé, Republic of Maldives; 3 Canadian Rivers Institute and Department of Biology, University of New Brunswick, Fredericton, New Brunswick, Canada; The Australian National University, Australia

## Abstract

Large numbers of the Globe Skimmer dragonfly (*Pantala flavescens*) appear in the Maldives every October–December. Since they cannot breed on these largely waterless islands, it has recently been suggested that they are “falling out” during a trans-oceanic flight from India to East Africa. In addition, it has been suggested that this trans-oceanic crossing is just one leg of a multi-generational migratory circuit covering about 14,000–18,000 km. The dragonflies are presumed to accomplish this remarkable feat by riding high-altitude winds associated with the Inter-tropical Convergence Zone (ITCZ). While there is considerable evidence for this migratory circuit, much of that evidence is circumstantial. Recent developments in the application of stable isotope analyses to track migratory dragonflies include the establishment of direct associations between dragonfly wing chitin *δ* ^2^H values with those derived from long-term *δ* ^2^H precipitation isoscapes. We applied this approach by measuring wing chitin *δ* ^2^H values in 49 individual *Pantala flavescens* from the November–December migration through the Maldives. Using a previously established spatial calibration algorithm for dragonflies, the mean wing *δ* ^2^H value of −117±16 ‰ corresponded to a predicted mean natal ambient water source of −81 ‰, which resulted in a probabilistic origin of northern India, and possibly further north and east. This strongly suggests that the migratory circuit of this species in this region is longer than previously suspected, and could possibly involve a remarkable trans-Himalayan high-altitude traverse.

## Introduction

Animal migrations are among the most spectacular phenomena in the natural world. Although research has focused on larger vertebrates, there are a number of examples of impressive long-distance migration among small insects. Perhaps the best known is that of the eastern Monarch Butterfly (*Danaus plexippus*) which completes a multi-generational migratory circuit between natal sites in Canada and the United States and wintering sites in Mexico [1]. It has been long suspected that several species of dragonfly also undertake long-distance annual migrations. One dragonfly species in particular, the Globe Skimmer or Wandering Glider (*Pantala flavescens*) is widely distributed throughout the tropics and many temperate areas, and is thought to undertake extensive migrations following the Inter-tropical Convergence Zone (ITCZ). This meteorological phenomenon is marked by a band of clouds around the globe near the equator, which moves north and south with the seasons. For a dragonfly, *Pantala flavescens* has a remarkably short larval life (34–43 d, [2], [3]), allowing it to breed in the ephemeral freshwater pools and floods produced by ITCZ rainfall. But even with such a brief larval life, when the adults emerge, the ITCZ has moved on. The adults are believed to migrate to the latitude where rains are falling by taking advantage of the converging air masses of the ITCZ. Indeed, *P. flavescens* has been categorized as an obligate ITCZ migrant [4]. However, any independent support of such continental-scale movements was lacking until it was proposed that *P. flavescens* completes a multi-generational migratory circuit encompassing India and eastern Africa [5]. That study documented regular movements of large numbers of this species through the Maldives during October-December, when individuals are presumed to be *en route* from India to east Africa. These movements coincide with favorable high-altitude winds from the north east associated with the ITCZ, and the trans-oceanic migration was supported by reports of arrival phenology of *P. flavescens* in southern India, Seychelles and East Africa [5]. However, supporting evidence remains largely circumstantial, and the small size of the insects precludes the use of transmitter technology to track population movements directly ([6], but see [7]). Fortunately, the measurement of intrinsic isotopic markers in metabolically inert tissue like wing chitin now allows the tracking of migratory insect populations through the use of continental patterns in stable isotope values of inorganic and organic substrates [8], [9].

We used stable-hydrogen isotope (deuterium, *δ* ^2^H) values in wing chitin of two samples of migrating *P. flavescens* that were collected in the Maldives (following “fall out” in December 2009 and November 2010) to infer their natal origins, based on well-known spatial patterns of *δ* ^2^H in precipitation and in major surface waters across the Indian subcontinent. *Pantala flavescens* arrives in Maldives across the ocean from southern India [5], presumably having been bred in ephemeral water bodies fed by the seasonal (June to September) monsoon rains of the subcontinent. We therefore expected our samples to reveal a southern or central Indian origin.

## Methods

### Ethics Statement

No specific permits were required for the described field studies. No specific permissions were required to collect dragonflies in the Maldives (dragonfly catching is a traditional and popular pastime in the Maldives; both samples were collected with the assistance of Maldivian scientists - acknowledged below). We confirm that neither collection site is protected. At one site (Malé), collection was made on public land. At the other site (Kunfunadhoo), collection was made in the grounds of a private resort hotel, with the approval and assistance of the resort environmental officer (Anke Hofmeister). The species collected (*Pantala flavescens*) is neither endangered nor protected. It is globally distributed and has been assessed by IUCN as Least Concern.

### Stable Isotope Analysis

Dragonflies were collected by RCA in the Maldives on Malé (4°11′N 73°31′E) between 7–9 November 2010 (n = 22) and on Kunfunadhoo Island in Baa Atoll (5°07′N 73°05′E) on 10 December 2009 (n = 27) during the annual migration event [5]. Specimens were euthanized by chloroform or freezing and stored dry in paper envelopes until shipment to the Stable Isotope Hydrology and Ecology Laboratory of Environment Canada in Saskatoon, Canada. All dragonfly wings were cleaned of surface oils in a 2∶1 chloroform:methanol solvent rinse and prepared for stable-hydrogen isotope analysis at the stable isotope laboratory [9]. Stable-hydrogen isotope analyses of dragonfly wings were conducted using the comparative equilibration method [10] and through the use of calibrated keratinous protein H reference materials. Stable-hydrogen isotope measurements were performed on H_2_ derived from high-temperature (1350°C) flash pyrolysis of 350 µg wing subsamples using continuous-flow isotope-ratio mass spectrometry. All results for non-exchangeable *δ* ^2^H are expressed in the typical delta notation, in units of per mil (‰), and normalized on the Vienna Standard Mean Ocean Water – Standard Light Antarctic Precipitation (VSMOW-SLAP) standard scale. Two keratin laboratory reference materials (CBS, KHS) were used to normalize the results, using the previously assigned *δ* ^2^H values of –197.0 and −54.1 ‰, respectively. A *δ* ^2^H calibration equation directly correlating measured dragonfly wing chitin (*δ* ^2^H_wing_) to precipitation-driven ambient natal water (*δ* ^2^H_p_) was derived by [9] who reported the relation *δ* ^2^H_wing_ = 0.91(*δ* ^2^H_p_) –42.54. We used this relationship to convert our measured *δ* ^2^H_wing_ values of *P. flavescens* to ambient natal water equivalent.

### Likelihood-based Assignment of Geographic Origins

We collated all available published surface water isotope data for India (Text S1, Table S1). We also compiled all available precipitation isotope data for the monsoonal months of July through September for this region as these would likely correspond to ephemeral water sources of a period appropriate for the short larval development phase of *P. flavescens* prior to adult arrival on the Maldives in November-December (Text S1, Table S2). Both surface water *δ* ^2^H and July-September monsoonal precipitation *δ* ^2^H were converted into isoscapes using simple kriging (Arcview 9.3, ESRI, Redlands, California, USA). We did not attempt an elevation-corrected model since the topography of India involves relatively low relief over much of the continent south of the Himalayas, with the only relatively negative precipitation *δ* ^2^H occurring in the mountains (Table S2).

**Figure 1:**
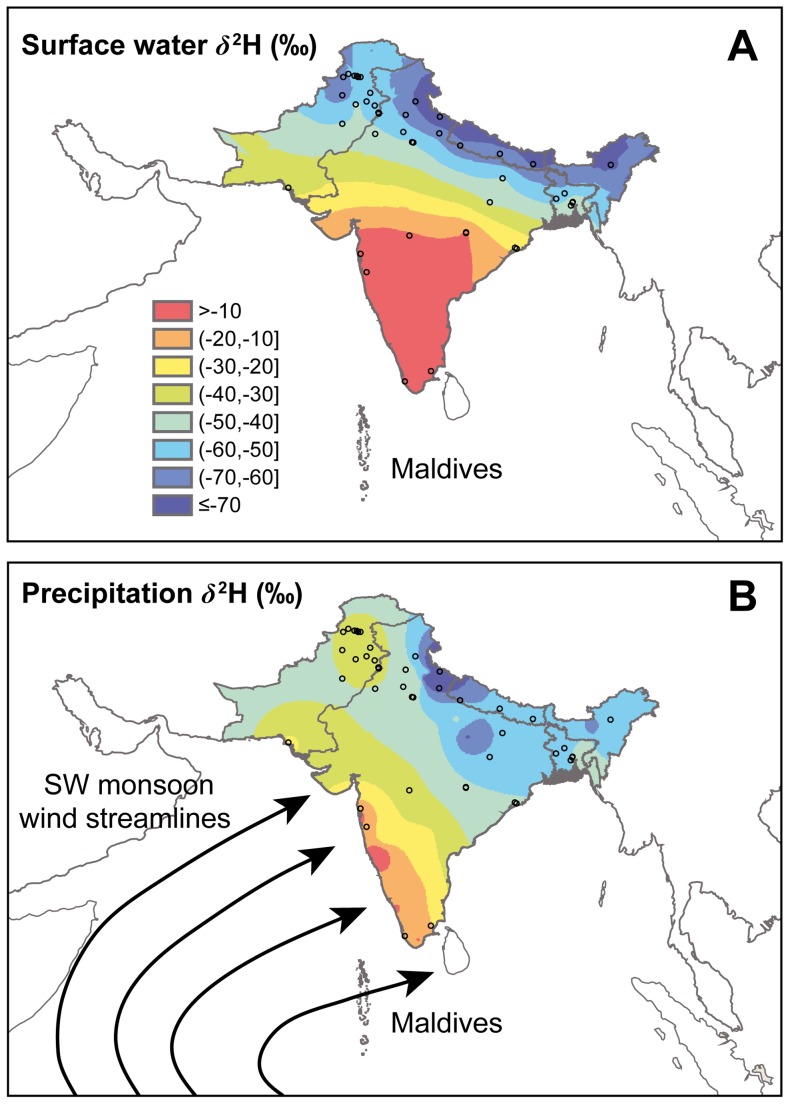
Predicted water *δ* ^2^H isoscapes for India. (a) surface water *δ* ^2^H (‰) and (b) precipitation amount-weighted *δ* ^2^H (‰) in monsoonal rainwaters (July-September)for the Indian subcontinent. (a) includes a schematic representation of typical southwest monsoon wind directions across the Arabian Sea during July.

A likelihood-based assignment method that incorporated estimates of uncertainty was used to link dragonfly wing *δ* ^2^H values from our Maldivian samples of *Pantala flavescens* to our surface water and precipitation *δ* ^2^H isoscapes for the Indian subcontinent (Figure 1). We used the likelihood-based methods described previously in [9]. The hydrological *δ* ^2^H isoscapes were first converted into a wing *δ* ^2^H isoscape by applying the calibration equation obtained from known-origin dragonfly wing values (*δ* ^2^H_wing_ = 0.91 *δ* ^2^H_p_ −42.54; [9]). This *δ* ^2^H wing isoscape was then imported into R Version 2.12.1 (R Development Core Team 2010) to assess the likelihood that any given cell within the *δ* ^2^H wing isoscape could represent the potential origin of each dragonfly individual using the normal probability density function. We used an expected mean wing *δ* ^2^H based on that predicted by the calibrated isoscape and a standard deviation of 19.1 ‰ estimated from the standard deviation of the residuals for the regression linking *δ* ^2^H_wing_ and *δ* ^2^H_p_ [9]. This provided a set of spatially explicit probability densities for each individual dragonfly. To assign all individuals to the natal basemap, we reclassified the spatially explicit probability densities into likely versus unlikely origins, by specifying an odds ratio. Based on 3∶1 odds that a given assigned dragonfly had really originated from a cell within that range, we identified the set of cells that defined the upper 75% of estimated ‘probabilities of origin’ and coded those as one, and all others as zero. Each dragonfly was assigned to multiple potential origins within the isoscape at the same time. The results of the assignment for all individuals were summed and mapped on the wing *δ* ^2^H isoscape to obtain the likely natal origin of the population where each cell then represented the number of dragonflies assigned to that location given our odds ratio.

## Results

**Figure 2:**
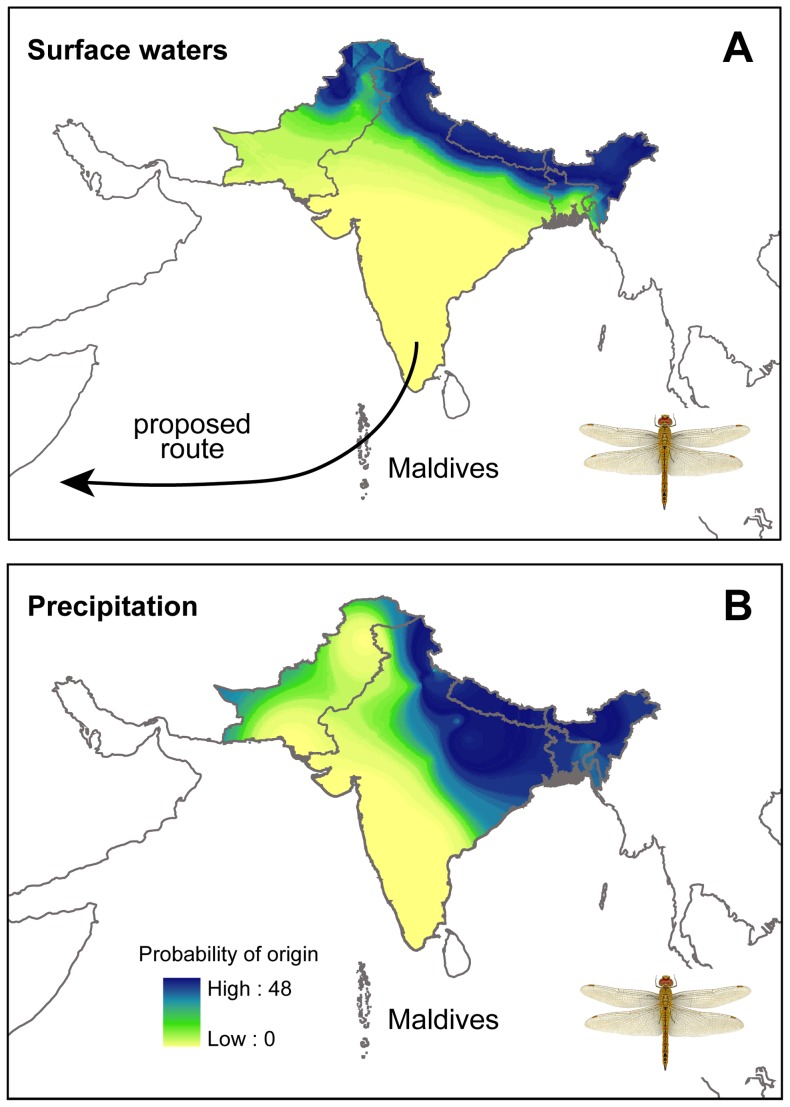
Predicted origins of *Pantala flavescens* intercepted on the Maldives. Depictions are based on the assumption of largely Indian origins for (a) surface water *δ* ^2^H (‰) and (b) precipitation amount-weighted *δ* ^2^H (‰) in monsoonal rainwaters (July-September) for the Indian subcontinent. The arrow in (a) gives a schematic indication of the proposed route of *P. flavescens* across the western Indian Ocean in about November. Inset: *Pantala flavescens*, courtesy of Forrest Mitchell. Potential isotopic origins not shown include regions north and east of that depicted here.

Both the surface water (Figure 1, Table S1,) and July-September monsoonal rain (Figure 1, Table S2) showed similar *δ* ^2^H patterns, with peninsular India generally having considerably higher *δ* ^2^H values compared to the low *δ* ^2^H values in the mountainous regions farther to the north. Our November sample of *P. flavescens* (*δ* ^2^H_wing_ = −115±17 ‰, n = 22, range −83 to −148 ‰) did not differ from the December sample (*δ* ^2^H_wing_ = −118±15 ‰, n = 27, range −89 to −151 ‰). The overall mean of −117±16 ‰ corresponded to predicted natal waters of −81 ‰ (range −118 to −44 ‰ [9]). Focusing largely on India for which our water isotope data were most complete, these results are consistent with our sample population of dragonflies originating at least as far north as northern India. Considering the range of the species and the nature of the precipitation isoscape for Asia, the sample of *P. flavescens* could have also derived from farther north and east along a similar band of latitude as the putative northern Indian origins. Such inferences of origin are conservative since any evaporation from ephemeral ponds would tend to isotopically enrich remaining waters in ^2^H, or the impression of more southerly origin. We depicted the potential origin in India of our population of dragonflies using a likelihood-based assignment procedure which confirmed northern origins based on a surface water and precipitation model (Figure 2).

## Discussion

*Pantala flavescens* arrive in Maldives during October-December, and are thought to originate in India [5]. We therefore predicted that our stable isotope results would confirm an Indian origin for our Maldivian samples of *P. flavescens*. While this prediction was indeed supported within the context of considering only possible Indian origins, both of our Maldives populations clearly originated from the far north of the subcontinent rather than from southern or central India.

This northern subcontinental origin is of interest for several reasons. First, the northern origin of *P. flavescens* arriving in Maldives extends the distance that they must be flying, not only to reach the Maldives, but also to complete the final leg of the intercontinental, multi-generational migratory circuit proposed by Anderson [5]. He suggested that these dragonflies used the seasonally alternating monsoon winds to follow the ITCZ, and breed in the short rains of equatorial East Africa in October–November; the summer rains of southern Africa in December–February; and the long rains of East Africa in March–May; before returning to India with the south-west monsoon to breed in June–July. Anderson [5] estimated that the distance covered (by perhaps four generations) in such a circuit would be of the order of 14000–18000 km. With our study suggesting that southwest monsoon breeding is concentrated at least as far as the north of the Indian subcontinent, those distances must be considered minima. The average distance covered may be closer to 18000 km, and some circuits possibly even longer. Furthermore, individual *P. flavescens* migrating from the northern subcontinent to East Africa via the Maldives must be flying in excess of 6000 km, including a trans-oceanic crossing of 3500 km. This is an extraordinary feat for a 5 cm long insect, and is, to the best of our knowledge, by far the longest regular single-generation migration documented for any insect.

Secondly, while the most parsimonious natal area for our samples of *P. flavescens* was northern India or Nepal, they could in fact have come from even further north and east. Certainly, from a regional perspective, wide areas of central Asia north of the Himalayas have mean annual precipitation *δ* ^2^H low enough to qualify as potential geographical origins for our samples as do the mountainous regions directly to the east of the Indian region we have depicted in Figure 2 ([11], http://www.waterisotopes.org). This raises the intriguing possibility that at least some *P. flavescens* might complete a trans-Himalayan migration. This species is known to fly higher than any other odonate [4], with several high-altitude records up to 6300 m in the Himalayan region [4], [12], [13].

Thirdly, our results raise an interesting question: if our two populations are representative, then why are *P. flavescens* arriving in Maldives in November-December from the far north of the subcontinent, and not central or southern India? One possible answer follows from Anderson’s [5] suggestion that the dragonflies appearing in Maldives in October-December are the offspring of individuals that crossed the Arabian Sea, from East Africa to the Indian subcontinent, in June–July. Those dragonflies are able to make that crossing with the aid of the southwest monsoon winds. But (due the effects of Coriolis force) only in northern India do these winds travel directly from East Africa; the southwest monsoon winds arriving on the southwest coast of India originate over the southern Indian Ocean ([14], Fig. 1a). Therefore *P. flavescens* might only be transported in large numbers from East Africa to northern Indian subcontinent, and not to central or southern India.

In conclusion, this study supports the hypothesis of Indian subcontinent origins of *P. flavescens* arriving in Maldives during November–December, and has extended the likely distances over which they migrate while recognizing that individuals may well be coming from natal origins even further north and east. We have further provided indirect support for the proposed migration of *P. flavescens* across the Arabian Sea from East Africa to northern Indian subcontinent during June–July. The use of stable isotope analyses to further unravel the behavior of these migratory dragonflies has been amply justified, and further studies of *P. flavescens* involving additional areas, seasons and isotopes are planned.

## Supporting Information

Table S1 **Summary of available surface water** ***δ*** **^2^H data for India.**
(DOC)

Table S2 **Summary of available amount-weighted precipitation** ***δ*** **^2^H for July-September for India and Pakistan.**
(DOC)

Text S1 **Precipitation and surface water** ***δ*** **^2^H summary**
(DOC)

## References

[1] Solensky MJ (2004) Overview of monarch migration. In: Oberhauser KS, Solensky MJ, editors. The Monarch Butterfly: Biology and Conservation. Ithaca: Cornell University Press. 79–83.

[2] Kumar A (1984) On the life history of *Pantala flavescens* (Fabricus) (Libellulidae: Odonata). Annals Entom. 2: 43–50.

[3] Suhling F, Schenk K, Paddefke T, Martens A (2004) A field study of the larval development in a dragonfly in African desert ponds (Odonata). Hydrobiologia 528: 75–85.

[4] Corbet PS (2004) Dragonflies: behavior and ecology of Odonata. Colchester: Harley Books. 829 p.

[5] Anderson RC (2009) Do dragonflies migrate across the western Indian Ocean? J Trop Ecol 25: 347–358.

[6] Hobson KA, Norris DR (2008) Animal migration: A context for using new techniques and approaches. In: Hobson KA, Wassenaar LI, editors, Tracking Animal Migration Using Stable Isotopes. London: Academic Press. 1–19.

[7] Wikelski M, Moskowitz D, Adelman JS, Cochran J, Wilcove DS, et al (2006) Simple rules guide dragonfly migration. Biol. Lett. 2: 325–329. 10.1098/rsbl.2006.0487 PMC1686212 17148394

[8] Hobson KA, Wassenaar LI (2008) Tracking Animal Migration Using Stable Isotopes. London: Academic Press. 144 p.

[9] Hobson KA, Soto DX, Paulson DR, Wassenaar LI, Matthews JH (2012) A dragonfly (δ^2^H) isoscape for North America: a new tool for determining natal origins of migratory aquatic emergent insects. Methods Ecol. Evol. 3: 766–772.

[10] Wassenaar LI, Hobson KA (2003) Comparative equilibration and online technique for determination of non-exchangeable hydrogen of keratins for use in animal migration studies. Isotopes Env. Health Stud. 39: 211–217. 10.1080/1025601031000096781 14521282

[11] Bowen GJ, Wassenaar LI, Hobson KA (2005) Application of stable hydrogen and oxygen isotopes to wildlife forensic investigations at global scales. Oecologia 143: 337–348. 15726429 10.1007/s00442-004-1813-y

[12] Vick GS (1989) List of the dragonflies recorded from Nepal, with a summary of their altitudinal distribution. Opuscula Zoologica Fluminensia 43: 1–21.

[13] Wojtusiak J (1974) A dragonfly migration in the high Hindu Kush (Afghanistan), with a note on high altitude records of *Aeshna juncea mongolica* Bartenev, and *Pantala flavescens* (Fabricius) (Anisoptera: Aeshnidae, Libellulidae). Odonatologica 3: 137–142.

[14] Fein JS, Stephens PL (1987) Monsoon. New York: John Wiley. 632 p.

