# Supplementary material for: Isotopic Evidence That Dragonflies (Pantala flavescens) Migrating through the Maldives Come from the Northern Indian Subcontinent
Source: PLoS One. 2012 Dec 20;7(12):e52594. doi: 10.1371/journal.pone.0052594 (PMC3527571; doi:10.1371/journal.pone.0052594)
Supplement: Table S1 — Summary of available surface water δ 2H data for India. (DOC) [file pone.0052594.s001.doc]

| **Project ID** | **Location** | **System type** | **δ^2^H (‰)** | **LAT** | **LONG** | **Source** |
| --- | --- | --- | --- | --- | --- | --- |
| UNDP-IND-73-008H | NW Himalayan springs | River | -77.0 | 32.00 | 77.50 | 1 (n=5) |
| UNDP-IND-73-008W | W coast springs | River | 3.6 | 18.00 | 73.50 | 1 (n=3) |
| UNDP-IND-73-008W | W coast springs | Lake | 8.6 | 19.50 | 73.00 | 1 (n=1) |
| UNDP/FAO-IND-81-010 | Haryana State | River | -62.5 | 29.50 | 76.50 | 1 (n=26) |
| UNDP/FAO-IND-81-010 | Haryana State | Lake | -57.7 | 30.90 | 76.70 | 1 (n=2) |
| RAS8084R-IND | Raipur city | River | -6.6 | 21.20 | 81.65 | 1 (n=3) |
| RAS8084R-IND | Raipur city | Lake | -2.3 | 21.26 | 81.65 | 1 (n=3) |
| RAS8084P-IND | Purna River basin | River | -6.8 | 21.00 | 77.00 | 1 (n=7) |
| RAS8092-IND | Tattapani area | River | -49.1 | 23.73 | 83.60 | 1 (n=1) |
| RAS8097D-IND | Landfill areas, Delhi | River | -67.1 | 28.63 | 77.33 | 1 (n=1) |
| RAS8097D-IND | Landfill areas, Delhi | Lake | -24.1 | 28.66 | 77.22 | 1 (n=4) |
| RAS8097B-IND | Ghazipur area | River | -37.8 | 25.70 | 84.65 | 1 (n=1) |
| RAS8104T-IND | Tiruvadanai aquifers | River | -8.5 | 9.88 | 78.78 | 1 (n=14) |
| RAS8104S-IND | Sasthamkotta lake | Lake | 2.9 | 9.03 | 76.63 | 1 (n=20) |
| IND-6234 | Tapoban and Badrinath | River | -96.0 | 30.75 | 79.50 | 1 (n=2) |
| IND-7746 | Lake Naini area | River | -53.7 | 29.38 | 79.46 | 1 (n=8) |
| IND-7904I | Indira Gandhi Canal | River | -45.4 | 29.33 | 74.20 | 1 (n=11) |
| IND-8398 | Delang-Puri Sector | River | -23.6 | 20.00 | 85.66 | 1 (n=5) |
| IND-8398 | Delang-Puri Sector | Lake | -16.0 | 19.92 | 85.80 | 1 (n=4) |
| RAS8097-BGD | Dhaka | River | -27.3 | 23.75 | 90.40 | 1 (n=15) |
| BGD-10068 | Dupi Tila aquifer | River | -25.1 | 23.71 | 90.38 | 1 (n=1) |
| RAS8084-PAK | Sheikhupura area | River | -58.3 | 31.66 | 74.17 | 1 (n=4) |
| RAS8097M-PAK | Multan area | River | -49.8 | 30.17 | 71.50 | 1 (n=3) |
| RAS8104P-PAK | Peshawar area | River | -74.4 | 34.00 | 71.58 | 1 (n=9) |
| RAS8104R-PAK | Rechna Doab | River | -59.7 | 32.00 | 73.50 | 1 (n=126) |
| RAS8104C-PAK | Chashma Nuclear Plant | River | -81.8 | 32.50 | 71.50 | 1 (n=159) |
| RAS8104T-PAK | Tarbela dam area | Lake | -85.1 | 34.08 | 72.70 | 1 (n=2) |
| RAS8104T-PAK | Tarbela dam area | River | -41.3 | 34.10 | 72.50 | 1 (n=6) |
| PAK-3620 | Mardan area | River | -50.9 | 34.25 | 72.00 | 1 (n=28) |
| PAK-4255 | Chaj Doab | Lake | -32.0 | 31.75 | 72.60 | 1 (n=5) |
| PAK-4255 | Chaj Doab | River | -47.6 | 32.70 | 73.80 | 1 (n=28) |
| PAK-4794 | Haripur area | Lake | -73.1 | 34.00 | 72.80 | 1 (n=53) |
| PAK-4794 | Haripur area | River | -21.8 | 34.00 | 73.00 | 1 (n=14) |
| PAK-9826 | Kasur area | Lake | -21.7 | 31.08 | 74.47 | 1 (n=11) |
| PAK-9826 | Kasur area | River | -83.2 | 31.00 | 74.50 | 1 (n=7) |
| PAK-11322 | Karachi coastal aq. | River | -35.1 | 24.92 | 67.08 | 1 (n=5) |
| PB79 | Karnali | Chisapani | -75.0 | 28.38 | 81.17 | 2 |
| PB60 | Narayani | Narayanganj | -73.0 | 27.70 | 84.43 | 2 |
| PB69 | Kosi | Chatra | -82.0 | 26.85 | 87.15 | 2 |
| AR15 | Brahmaputra | Tezpur | -85.0 | 26.80 | 93.52 | 2 |
| BR417 | Ganges | Harding Bridge | -54.0 | 24.02 | 89.04 | 2 |
| BR402 | Brahmaputra | Sirajganj | -67.0 | 24.46 | 89.73 | 2 |
| BR720 | Padma | Mawa | -63.0 | 23.46 | 90.25 | 2 |
