# Supplementary material for: Isotopic Evidence That Dragonflies (Pantala flavescens) Migrating through the Maldives Come from the Northern Indian Subcontinent
Source: PLoS One. 2012 Dec 20;7(12):e52594. doi: 10.1371/journal.pone.0052594 (PMC3527571; doi:10.1371/journal.pone.0052594)
Supplement: Table S2 — Summary of available amount-weighted precipitation δ 2H for July-September for India and Pakistan. (DOC) [file pone.0052594.s002.doc]

| **Sample Site Name** | **Latitude** | **Longitude** | **Altitude** | **Period** | **Average δ^2^H (‰)** | **Source** |
| --- | --- | --- | --- | --- | --- | --- |
| TIRUNELVELI | 8.727778 | 77.718333 | 4 | 2003/2004 | -9.0 | 4 |
| KOZHIKODE (CALICUT) | 11.25 | 75.78 | 20 | 1997-2007 | -8.2 | 5 |
| BANGLORE | 12.971667 | 77.594167 | 897 | 2003/2004 | -15.8 | 4 |
| BELGAUM | 15.880556 | 74.493333 | 747 | 2003/2004 | 2.9 | 4 |
| KAKINADA | 17.021111 | 74.846111 | 8 | 2003-2006 | -27.1 | 4 |
| HYDERABAD | 17.45 | 78.47 | 545 | 1997-2000 | -27.2 | 3 |
| MUMBAI | 18.9 | 72.82 | 10 | 1961-1966; 1973-1977 | -3.9 | 3 |
| KOLKATA | 22.797778 | 88.371667 | 6 | 2004-2006 | -53.3 | 4 |
| SAGAR | 23.826111 | 78.7625 | 551 | 2003-2006 | -42.7 | 4 |
| MAWLONG* | 25.219167 | 91.662778 | 915 | 2007-2008 | -47.5 |  |
| ALLAHABAD | 25.45 | 81.733333 | 98 | 2008 | -71.9 | 3 |
| SHILLONG | 25.57 | 91.88 | 1598 | 1969, 1976 | -59.6 | 3 |
| GUWAHATI | 26.190833 | 91.795278 | 54 | 2004 | -63.3 | 4 |
| LUCKNOW | 26.874722 | 80.938889 | 128 | 2004 | -48.0 | 4 |
| NEW DELHI | 28.58 | 77.2 | 212 | 61-69,73-76,78-81,83-88,91-95,2000-2008 | -45.8 | 3 |
| ROORKEE | 29.867778 | 77.893889 | 274 | 2004-2006 | -45.7 | 4 |
| RISHIKESH | 30.112222 | 78.3025 | 356 | 2005-2006 | -80.6 | 4 |
| DEVPRAYAG | 30.140556 | 78.596667 | 465 | 2004-2006 | -86.5 | 4 |
| TEHRI | 30.352778 | 78.483333 | 640 | 2004-2006 | -88.3 | 4 |
| UTTARKASHI | 30.729167 | 78.446667 | 1140 | 2004-2006 | -52.7 | 4 |
| GOMUKH | 30.926111 | 78.940278 | 3800 | 2004-2006 | -90.6 | 4 |
| DOBRANI* | 30.946111 | 78.688056 | 2050 | 2004-2006 | -68.1 | 4 |
| GANGOTRI | 30.996667 | 78.940278 | 3053 | 2004-2006 | -118.4 | 4 |
| JAMMU (INDIAN CONTROLLED KASHMIR) | 32.6925 | 74.846111 | 367 | 2003-2006 | -37.4 | 4 |
| KARACHI | 24.9 | 67.13 | 23 | 1961-1975 | -26.4 | 3 |
| ISLAMABAD-NILORE | 33.656944 | 73.266111 | 575 | 1992-2006 | -31.4 | 3 |
|  | | | | | | |
