# Supplementary material for: Isotopic Evidence That Dragonflies (Pantala flavescens) Migrating through the Maldives Come from the Northern Indian Subcontinent
Source: PLoS One. 2012 Dec 20;7(12):e52594. doi: 10.1371/journal.pone.0052594 (PMC3527571; doi:10.1371/journal.pone.0052594)
Supplement: Text S1 — Precipitation and surface water δ 2H summary (DOC) [file pone.0052594.s003.doc]

- 1. Precipitation and surface water *δ*^2^H summary

1.1 Surface water *δ*^2^H

We collated all available published data on surface waters in India (Table S1). Most of these records were summarized in the International Atomic Energy Agency (IAEA) atlas [1] with the addition also of [2]. These spatially explicit data were then depicted using simple kriging (ArcGIS 9.3, ESRI, Redlands, California, USA; Figure 1).

- 1. July-September monsoonal *δ*^2^H

All available precipitation *δ*^2^H data for India was obtained from [3]. Precipitation amount-weighted monthly (July-September) *δ*^2^H were averaged for all available years (Table S2). These spatially explicit data were then depicted using simple kriging (ArcGIS 9.3, ESRI, Redlands, California, USA; Figure 1).

**References**

1 International Atomic Energy Agency (2008) Atlas of isotope hydrology - Asia and the Pacific. Vienna: IAEA.

2 Galy V, Eglinton T, France-Lanord C, Sylva S (2011) The provenance of vegetation and environmental signatures encoded in vascular plant biomarkers carried by the Ganges–Brahmaputra rivers. Earth Planet. Sci. Lett. 304: 1-12.

3 Global Network for Isotopes in Precipitation (GNIP) database. Vienna: International Atomic Energy Agency.

4 Kumar B, Rai SP, Saravana Kumar U, Verma SK, Garg P, Vijaya Kumar SV et al. (2010) Isotopic characteristics of Indian precipitation. Water Resour. Res. 46: W12548, doi:10.1029/2009WR008532.

5 Warrier CU, Babu MP, Manjula P, Velayudhan KT, Hameed AS, Vasu K (2010) Isotopic characterization of dual monsoon precipitation – evidence from Kerala, India. Current Sci. 98: 1487-1495.

6 Breitenbach SFM, Adkins JF, Meyer H, Marwan N, Kumar KK, Haug GH (2010) Strong influence of water vapor source dynamics on stable isotopes in precipitation observed in Southern Meghalaya, NE India. Earth Planet. Sci. Lett. 292: 212-220.
